# Inhibitors of Bacterial Extracellular Vesicles

**DOI:** 10.3389/fmicb.2022.835058

**Published:** 2022-02-23

**Authors:** Jianwei Chen, Hongfang Zhang, Siqi Wang, Yujie Du, Bin Wei, Qiang Wu, Hong Wang

**Affiliations:** ^1^College of Pharmaceutical Science and Collaborative Innovation Center of Yangtze River Delta Region Green Pharmaceuticals, Zhejiang University of Technology, Hangzhou, China; ^2^State Key Laboratory of Quality Research in Chinese Medicines, Macau University of Science and Technology, Taipa, Macau SAR, China; ^3^Key Laboratory of Marine Fishery Resources Exploitment and Utilization of Zhejiang Province, Zhejiang University of Technology, Hangzhou, China

**Keywords:** extracellular vesicles, outer membrane vesicles, antibacterial activity, antibiotics resistance, membrane vesicles

## Abstract

Both Gram-positive and Gram-negative bacteria can secrete extracellular vesicles (EVs), which contain numerous active substances. EVs mediate bacterial interactions with their hosts or other microbes. Bacterial EVs play a double-edged role in infections through various mechanisms, including the delivery of virulence factors, modulating immune responses, mediating antibiotic resistance, and inhibiting competitive microbes. The spread of antibiotic resistance continues to represent a difficult clinical challenge. Therefore, the investigation of novel therapeutics is a valuable research endeavor for targeting antibiotic-resistant bacterial infections. As a pathogenic substance of bacteria, bacterial EVs have gained increased attention. Thus, EV inhibitors are expected to function as novel antimicrobial agents. The inhibition of EV production, EV activity, and EV-stimulated inflammation are considered potential pathways. This review primarily introduces compounds that effectively inhibit bacterial EVs and evaluates the prospects of their application.

## Introduction

Extracellular vesicles (EVs) are vesicles with double-layer membrane structures, which are secreted from the cell membrane (Cheng and Schorey, 2019; Briaud and Carroll, 2020). According to the size of vesicles, EVs are divided into various subgroups, including microvesicles, exosomes, and apoptotic bodies (Peng et al., 2020). Moreover, EVs carry various active substances (e.g., proteins, lipids, lipoproteins, DNA, and RNA), which play an important role in intercellular communication, immune modulation, and the spread of infections. EV membranes protect the encapsulated molecules from the degradation of numerous enzymes (Ohno et al., 2016).

Bacterial EVs were first identified in the Gram-negative bacteria, *Escherichia coli* (*E. coli*) (Hoekstra et al., 1976). Later, Lee et al. (2009a) found that *Staphylococcus aureus* (*S. aureus*) naturally secrete vesicles into the extracellular milieu. Further characterizations revealed that both their densities and sizes are similar to those of Gram-negative bacteria (Lee et al., 2009a). Gram-positive-associated EVs are termed membrane vesicles (MVs), whereas Gram-negative bacteria secreted EVs are termed outer membrane vesicles (OMVs) due to their origin of the outer membrane (Kim et al., 2013; Bitto and Kaparakis-Liaskos, 2017). Previous studies have shown the functions of bacterial vesicles include the acquisition of nutrients, adhesion to hosts, delivery of virulence factors, and immune modulation (Jan, 2017). These vesicles also participate in various physiological and pathological processes of the bacteria and host (Ellis and Kuehn, 2010; Brown et al., 2015). Furthermore, the EVs of various pathogenic bacteria contribute to potential virulence by transferring bacterial components over long distances, superseding direct bacterial connections with their host (Unal et al., 2011).

**FIGURE 1:**
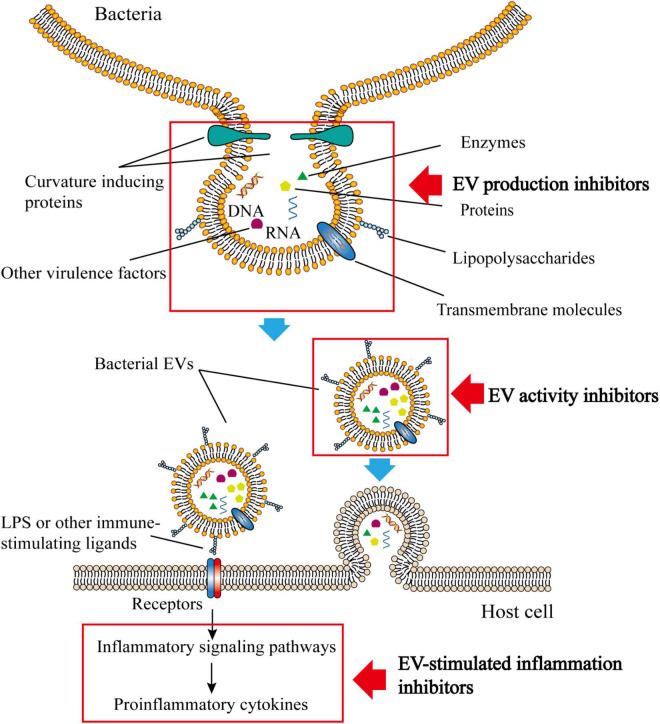
Structure, composition and function of bacterial EVs. Bacterial EVs carry various active substances and can be transferred into host cells by endocytosis. The immunogenicity of bacterial EVs is sufficient to stimulate the immune inflammatory response of the host. Inhibitors of bacterial vesicles typically target the production of bacterial EVs, their activity, or the inflammatory response that bacterial vesicles stimulate.

Due to the unique properties of bacterial EVs, they have gained increased attention as novel causative agents in infections and thus represent potential targets for the identification of new antimicrobial substances. Therefore, this review summarizes the compounds which have been shown to display an inhibitory effect on bacterial EVs. These inhibitors typically target EV production or their efficacy in bacterial invasion of the host (Figure 1).

## The Functions of Bacterial Extracellular Vesicles

Bacteria harbor various virulence factors, including bacterial enzymes, biofilms, biosurfactant, capsules, siderophores, and other structural materials (Chen et al., 2017, 2019, 2020). Several studies have demonstrated that bacterial EVs carry and deliver virulence factors to improve bacterial proliferation and survival within the host (Sharma et al., 2016; Leitao, 2020). Nakao et al. (2014) reported that the oral pathogens *Porphyromonas gingivalis* (*P. gingivalis*) release lipopolysaccharides (LPS) and gingipains in their EVs, causing epithelial tissue destruction in dental disease (Nakao et al., 2014). *Bacillus anthracis* (*B. anthracis*) produce EVs that contain biologically active virulence factors that are toxic to macrophages (Rivera et al., 2010). Notably, bacterial EVs also deliver genetic materials. Periodontal pathogenic bacteria like *Aggregatibacter actinomycetemcomitans* (*A. actinomycetemcomitans*) and *Treponema denticola* (*T. denticola*) transport msRNAs (small RNAs similar in size to miRNAs) via EVs. These msRNAs most likely act as bacterial signaling molecules that are associated with interactions between periodontal pathogens and the host (Choi et al., 2017). In addition, substances that promote bacterial invasion have also been identified in some bacterial EVs. *Mycobacterium tuberculosis* (*M. tuberculosis*)-derived EVs carry heparin-binding hemagglutinin adhesion (HBHA) and antigen Ag85 complex (Ag85), which enhance bacterial adhesion to host cells (Lee et al., 2015).

As derivatives of heterogeneous pathogens, EVs are capable of triggering both innate and acquired immune responses. This is because bacterial EVs are loaded with various immune-stimulating ligands (e.g., LPS, peptidoglycan, lipoprotein, and bacterial nucleic acids) which can be identified by pathogen recognition receptors and activate the host immune system (Kaparakis-Liaskos and Ferrero, 2015). Yang et al. (2020) found that flagellated bacteria, *Pseudomonas aeruginosa* (*P. aeruginosa*) and *Salmonella*, secreted EVs that stimulated the NLRC4 inflammasome pathways via flagellin-mediated transfer to the host cytoplasm. *Moraxella catarrhalis* (*M. catarrhalis*) EVs induce B cell activation via the TLR2 and TLR9 pathways (Vidakovics et al., 2010). *P. aeruginosa*-derived exosomes up-regulate regulatory T cell responses and down-regulate Th2 responses in an allergic immune response (Ding et al., 2018). Bacterial EVs also affect other immune cells, including macrophages, neutrophils, natural killing (NK) cells, and dendritic cells (DCs) (Kaparakis-Liaskos and Ferrero, 2015; Zhang et al., 2018).

Bacterial EVs may cause immune escape, which involves several different mechanisms. *Pneumococci*-produced EVs help the bacteria evade complement-mediated opsonophagocytosis via binding to complement proteins (Codemo et al., 2018). One of the key factors associated with neutrophil-mediated immune defenses is neutrophil extracellular traps (NETs), which attach to pathogens and allow neutrophils to kill them (Papayannopoulos, 2017). *Streptococcus pneumoniae* (*S. pneumoniae*) EVs contain tatD, a type of endodeoxyribonuclease that displays NET degrading activity (Jhelum et al., 2018). Moreover, *M. tuberculosis* EVs impair the ability of antigen presentation via inhibiting major histocompatibility complex-II (MHC-II) expression (Singh et al., 2011; Jurkoshek et al., 2016; Athman et al., 2017). In addition, pathogen EVs are also able to promote the apoptosis of immune cells (Winter et al., 2014; Avila-Calderon et al., 2015).

Recently, bacterial EVs have been found to mediate antibiotic resistance. *Acinetobacter baumanii* (*A. baumanii*) EVs release Oxacillinase (OXA)-58, a D β-lactamases that hydrolyzes carbapenem to protect carbapenem-susceptible bacteria against carbapenem killing (Liao et al., 2015). Similarly, *E. coli* MG1655 releases EVs that protect *E. coli* from the killing of colistin and melittin (Kulkarni et al., 2015). *M. catarrhalis* EVs release β-lactamase, which promotes their survival and even the survival of other symbiotic pathogens [e.g., *Hemophilus influenzae* (*H. influenza*) and *S. pneumoniae*] (Schaar et al., 2011). Some bacterial EVs also transmit specific genes to other bacteria, thereby mediating antibiotic resistance (Rumbo et al., 2011).

Bacteria participate in the interactions of bacterial community and environment via the secretion of EVs. Studies have demonstrated that several Gram-positive bacteria kill other competing microbes by secreting EVs (Yu et al., 2018). EVs from *Burkholderia thailandensis* (*B. thailandensis*) contain antimicrobial compounds, such as peptidoglycan hydrolases, rhamnolipid and 4-hydroxy-3-methyl-2-(2-nonenyl)-quinoline (HMNQ). These substances inhibit methicillin-resistant *S. aureus* (MRSA) to improve the survival of *B. thailandensis* (Wang et al., 2020). *Myxococcus xanthus* (*M. xanthus*) EVs hunt other microbes by transmitting hydrolytic enzymes and other antimicrobial substances (Berleman et al., 2014).

Moreover, nucleic acid substances in bacterial EVs can be transferred horizontally to host cells to regulate host gene expression (Ahmadi Badi et al., 2020). EVs also enhance the biofilm formation to protect pathogens against antibiotics and host immunity (Jarzab et al., 2020; Seike et al., 2020). Bacteria can use EVs to exchange cell surface substances and improve their survival during mammalian infections (Zingl et al., 2020). They also play a role in nutrient acquisition (e.g., iron), which is crucial for bacterial growth and reproduction (Prados-Rosales et al., 2014; Yu et al., 2018).

As a key factor involves in the interaction between bacteria and hosts, bacterial EVs exhibit double-edged effects in hosts. While EVs promote bacterial invasion of hosts, they also modulate immune responses and suppress other microbes. Together, these interactions can help us to understand the novel mechanisms of bacterial invasion and develop more effective therapies which can be used to overcome pathogenic infections.

## Inhibitors of Bacterial Extracellular Vesicles

As mentioned above, bacterial EVs carry various virulence factors, which play a significant role in bacterial infections. From this perspective, the modulation of bacterial EVs represents a potential target for regulating bacterial invasion. The compounds which suppress the release of EVs or block their functions are expected to be developed as effective drugs against bacterial infectious diseases.

### Inhibitors of Suppressing Extracellular Vesicle Production

Bacterial EVs contain various pathogenic factors and the inhibition of EV secretion has been shown to reduce the spread of toxic compositions. Compounds typically inhibit EV production via suppressing EV formation signaling pathways.

#### Pseudomonas Quinolone Signal Inhibitors

*P. aeruginosa* secretes the signal molecule, 2-heptyl-3-hydroxy-4-quinolone, termed the Pseudomonas quinolone signal (PQS). PQS is known as a quorum-sensing (QS) molecule in *P. aeruginosa* which is involved in the production of several bacterial virulence factors and biofilm formation (Pesci et al., 1999; Diggle et al., 2003). The production of PQS is initiated with anthranilic acid and involves various enzymatic reactions (Sabir et al., 2020). As a positive regulator of EV production, PQS induces EV production by interacting with the acyl chains and 4-phosphate of bacterial LPS (Mashburn-Warren et al., 2008). Besides PQS, bacteria secrete several other bicyclic compounds as extracellular signals. For example, indole acts as a QS signal molecule in *E. coli*. Indole compounds, including indole and 7-hydroxyindole (7HI), are able to inhibit enterohemorrhagic *E. coli* biofilms and reduce *P. aeruginosa* virulence (Lee et al., 2007, 2009b). Several bacteria also convert indole into oxidized products (e.g., hydroxyindoles, isatin, and indigo). These oxidized products may play the same role as indoles in influencing the production of EVs. Therefore, many bicyclic compounds, including indole derivates exist in the bacterial environment and play a significant role in bacterial communications (Rui et al., 2004; Yin et al., 2005).

**FIGURE 2:**
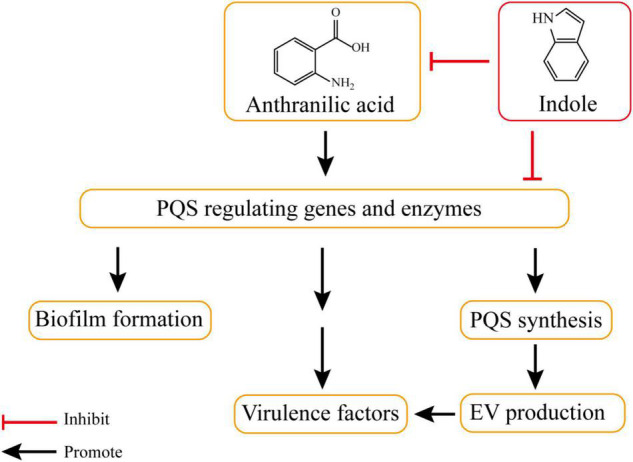
Indole inhibits PQS synthesis and EV production. The production of PQS is initiated with anthranilic acid. PQS involves in the production of several bacterial virulence factors and biofilm formation, and it positively regulates the production of EV through gene regulation or enzymatic reactions. Indole can inhibit EV production by involving in the synthesis or the degradation of anthranilic acid and affecting the activation of PQS synthesis-related enzymes.

Indole (**1**) and its oxidation products, such as 4HI (4-hydroxyindole) (**2**), 5HI (5-hydroxyindole) (**3**), 6HI (6-hydroxyindole) (**4**), oxidole (**5**) and isatin (**6**), can significantly inhibit EV production in *P. aeruginosa* (Table 1). Compared with a control, 500 μM of **1** led to a 92% reduction in EV production. Products **2**, **3**, **4**, and **6** also showed similar inhibitory efficacy with indole. In addition, product **5** led to a 55% decrease in EVs. PQS synthesis was also reduced in the presence of these indole derivative compounds, suggesting that the reduction of EVs might be related to the inhibition of PQS synthesis. Further investigation indicated that indole suppressed EV production via regulating PQS rather than inhibiting the interaction between PQS and LPS. To determine whether the reduction of PQS syntheses was due to the inhibited expression of the PQS biosynthetic operon, the expression of related genes (e.g., *pqsABCDE* and the *pqsA* promoter) was examined. The presence of 500 mM indole led to a decrease in *pqsABCDE* expression; however, it did not affect the autoregulation of *pqsABCDE* by PQS. The presence of indole did not change *pqsH* transcription, suggesting that *pqsH* is not related to the inhibition of PQS synthesis by indole. Moreover, the efficacy of the *pqsA* promoter was also inhibited in the presence of indole derivative products, indicating that these compounds suppressed PQS-stimulated transcription. Two hypotheses were considered regarding the mechanism by which indole suppresses PQS synthesis: (1) Indole was involved in the synthesis or the degradation of anthranilic acid; and (2) Indole affected the activation of any PQS synthesis-related enzymes after transcription (Figure 2; Tashiro et al., 2010).

**TABLE 1:** EV production inhibitors that targeting PQS.

No.	Name	Structure	Target	EV production inhibition (%) at 500 μM in *P. aeruginosa*	References
1	Indole	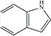	PQS	92	
2	4HI	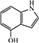	PQS	92	
3	5HI	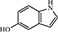	PQS	85	
4	6HI	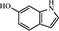	PQS	90	
5	Oxidole	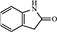	PQS	55	
6	Isatin	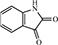	PQS	90	
					Tashiro et al., 2010
7	8-quinolinole	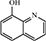	PQS	88	
8	Naphthalene	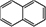	PQS	44	
9	1-Naphthol	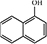	PQS	88	
10	2-Naphthol	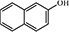	PQS	84	
11	2,3-Dihydroxynaphthalene	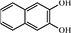	PQS	90	
12	1-Aminonaphthalene	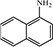	PQS	80	

To investigate whether the bicyclic structure was critical for the inhibition of EVs and PQS, other cyclic compounds, including 8-quinolinole (**7**), naphthalene (**8**), 1-Naphthol (**9**), 2-Naphthol (**10**), 2,3-Dihydroxynaphthalene (**11**), 1-Aminonaphthalene (**12**), catechol and carbazole were tested. According to the results, catechol and carbazole did not suppress EV production or PQS synthesis. Compound **8** led to a 44% reduction in EV production and other naphthalene analogs significantly repressed both EV production and PQS synthesis (Table 1). These findings suggested that bicyclic compounds, rather than monocyclic or tricyclic compounds, inhibited EV production and PQS synthesis (Tashiro et al., 2010).

#### Peptidyl Arginine Deiminase Inhibitors

Peptidyl arginine deiminases (PADs) are a series of calcium-activated enzymes that participate in the post-translational deamination or citrullination of arginine residues to citrulline, leading to structural and functional alterations in target proteins. PADs have been preserved throughout various phylogeny, ranging from microbes to mammals (Magnadóttir et al., 2018). Previous studies have established that EV secretion is mainly PAD-driven, can be effectively suppressed using PAD inhibitors, and that such suppression sensitized cancer cells to chemotherapy (Kosgodage et al., 2017, 2019b). Therefore, it was investigated if this might be a phylogenetically conserved mechanism in bacterial EV secretion and can be utilized to sensitize bacteria to antibiotics.

The effect of some PAD-specific inhibitors on EV secretion and antibiotic sensitivity of *E. coli* VCS257 and *S. aureus* subsp. *aureus Rosenbach* was analyzed. In *E. coli* VCS257, the PAD4-specific inhibitor, GSK199 (**13**) exhibited a 66.4% reduction in EV production. BB-Cl-amidine (**14**) and Cl-amidine (**15**) led to a 53.8% reduction and a 42.4% reduction in EV production, respectively. The PAD2-specific inhibitor AMF30a (**16**) exhibited a 28.2% decrease in EV production. In the Gram-positive bacterium *S. aureus* subsp. *Sureus Rosenbach*, **13** led to a 22.5% reduction of EV production, whereas **16** showed only a 3.4% decrease in EV production. Treatment with **14** and **15** showed 7.6 and 12.5% inhibition in EV production, respectively (Table 2). The difference between Gram-positive and Gram-negative bacteria indicate that the composition of their membrane plays a role in EV formation. The thickened peptidoglycan cell wall of Gram-positive bacteria limited the penetration of drugs in to the cells. However, there is a thin layer of peptidoglycan of Gram-negative cell membrane, the high lipid content of them may facilitate the penetration of lipid soluble drugs and lead to a more effective response. In addition, some changes in the EV profile were discovered following treatment with the PAD inhibitors, which varied between the inhibitors that were used. Furthermore, the PAD inhibitors could be utilized to improve the sensitivity of selected antibiotics. Interestingly, while **13** was overall the most potent EV inhibitor, the pan-PAD inhibitors (**14** and **15**) exhibited a similar trend and could occasionally elicit a stronger sensitivity to the antibiotic. This may due to their disparity in hydrophobicity, which plays a role in the uptake and penetration of cells, affecting EV release (Kosgodage et al., 2019b).

**TABLE 2:** EV production inhibitors that targeting PADs.

No.	Name	Structure	Target		Activity	References
				***E. coli* VCS257**	***S. aureus* subsp. *sureus* Rosenbach**	
13	GSK199	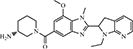	PADs	Reduce 66.4% EV production at 10 μM	Reduce 22.5% EV production at 10 μM	
14	BB-Cl-amidine	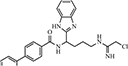	PADs	Reduce 53.8% EV production at 5 μM	Reduce 7.6% EV production at 5 μM	
						Kosgodage et al., 2019b
15	Cl-amidine	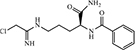	PADs	Reduce 42.4% EV production at 50 μM	Reduce 12.5% EV production at 50 μM	
16	AMF30a	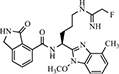	PADs	Reduce 28.2% EV production at 5 μM	Reduce 3.4% EV production at 5 μM	

#### Other Extracellular Vesicle Production Inhibitors

Sigma factor B (SigB) is a positive auto-regulatory global regulator that participates in the general stress response via directly or indirectly affecting gene expression. SigB is also involved in the bacterial secretion system by producing EVs (Depke et al., 2012; Lee et al., 2013). *S. aureus* was found to produce EVs that were able to deliver virulent bacterial components to host cells and lead to cell apoptosis (Gurung et al., 2011). In view of these problems, targeting natural compounds for their anti-SigB activity could be considered as a treatment for MRSA infection. Rhodomyrtone (**17**), a type of acylphloroglucinol, was extracted from Rhodomyrtus tomentosa (Aiton) Hassk (Saising et al., 2018). It was previously found to exhibit strong antibacterial activity in various Gram-positive bacteria including *S. aureus* and it was related to SigB activity (Limsuwan et al., 2009). Further, the effects of **17** on EV release were performed. **17** could inhibit EV production during the exponential growth phase of bacteria. A reduction in the amount of EVs was highly significant in the clinical isolate strain of *S. aureus*, as EVs were decreased by 86.7 ± 3.8% when treated with 0.5 × MIC of **17** (0.25 μg/mL) (Table 3). It was also found that **17** treatment significantly decreased SigB activity in *S. aureus*. The SigB activity of *S. aureus* was reduced by approximately fivefold following the treatment with 0.5 × MIC of **17** for 3 h. Further investigation indicated that sigB-related genes (e.g., *sigB* and *clfA*) were downregulated, whereas *clpL*, another sigB-related gene, was up-regulated. These changes of sigB-dependent genes were associated with SigB activity. These findings supported the finding that **17** suppressed *S. aureus* SigB activity, resulting in the reduction of EV production. Interestingly, when the bacteria reached the early stationary phase or late stationary phase, sigB activity increased. This observation suggests that the inhibitory efficacy of **17** depends on the bacterial growth phase. Moreover, it was believed that **17** induced a stress response via activating SigB instead of directly affecting SigB activity (Mitsuwan et al., 2019).

**TABLE 3:** Other EV production inhibitors.

No.	Name	structure	Target	Activity	References
**17**	Rhodomyrtone	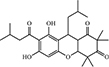	SigB	Reduce 86.7% EV production at 0.25 μg/mL in *S. aureus*	Mitsuwan et al., 2019
**18**	CBD	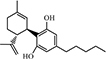	Unknown	Reduce 73% EV production at 1 μM in *E. coli*	Kosgodage et al., 2019a
**19**	FOM	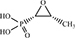	NLRP3 MAPKs	Reduce 92% EV production at 4 μg/mL in *S. aureus*	An et al., 2019

Cannabidiol (CBD) (**18**) is a phytocannabinoid extracted from *Cannabis sativa* and exhibits anti-inflammatory and anti-bacterial properties (Hernández-Cervantes et al., 2017; Kovalchuk and Kovalchuk, 2020; Blaskovich et al., 2021). Previous studies identified **18** as an effective inhibitor of EV release in eukaryotes, and therefore, it was sought to determine whether **18** was involved in bacterial EV release (Kosgodage et al., 2018). The study by Kosgodage et al. (2018) showed that **18** had a pronounced inhibitory effect on the total EV secretion from *E. coli* VCS257 (Table 3). The lower dose displayed an even stronger inhibitory efficacy. Treatment with 1 μM **18** led to a 73% reduction in EV release whereas 5 μM **18** led to a 54% reduction. However, contrary to *E. coli*, 5 μM **18** treatment had no significant inhibitory effect on EV release from the Gram-positive *S. aureus* subsp. *aureus Rosenbach*. In addition, **18** treatment altered the EV sizes and protein profiles in *E. coli* VCS257. It was identified that various proteins, including proteins related to metabolism, cellular respiration, and antibiotic metabolic processes, which differ in CBD-treated *E. coli* VCS257 released EVs, compared to non-treated *E. coli* VCS257-released EVs. The effects of CBD on proteins involved in antibiotic metabolism might contribute to its antibiotic sensitization. Indeed, CBD-mediated EV suppression enhanced the antibacterial effects of erythromycin, vancomycin, rifampicin, and kanamycin in *E. coli* VCS257 (Kosgodage et al., 2019a). Thus, as an effective inhibitor of bacterial EVs, **18** may have potential as a putative adjuvant agent for co-application with antibiotics to reduce antibiotic resistance.

α-Hemolysin (Hla) was contained by staphylococcal EVs, which play a significant role in staphylococcal infections. Fosfomycin (FOM) (**19**) was an antibiotic that was bactericidal both *in vitro* and *in vivo*, especially against MRSA (Poeppl et al., 2011). The study by An et al. (2019) found that a sub-inhibitory concentration of **19** showed an inhibitory effect on the release of Hla and protected mice against *S. aureus* EV-induced pneumonia. Further *in vitro* studies observed that treatment with **19** significantly suppressed EV release from *S. aureus* and the level of Hla secreted from *S. aureus* EVs. The results implied that Hla was associated with *S. aureus* EVs, and **19** inhibited the release of *S. aureus* EVs and EV-contained Hla. Pretreated with 4 μg mL^–1^ **19** decreased almost 92% production of EVs in strain 8325-4 of *S. aureus*. Further investigation demonstrated that **19** inhibited the activation of NLRP3 and MAPK inflammasomes, which were stimulated by Hla from staphylococcal EVs, thereby reducing the inflammation caused by *S. aureus* EVs and Hla. Compared to the untreated group, the level of IL-1β and IL-18 stimulated by EVs from *S. aureus* strain 8325-4 was decreased by approximately 40 and 72%, respectively, after **19** pretreatment. Therefore, **19** was considered to represent a promising clinical medication because it could both kill pathogens, as well as inhibit their pathogenicity (An et al., 2019).

### Inhibitors of Blocking the Inflammatory Response Stimulated by Extracellular Vesicles

Bacterial EVs are able to mediate serious inflammatory responses. Molecules that reduce the release of bacterial EV-induced pro-inflammatory factors have the potential to be developed into antibacterial drugs. Major inflammatory factors include interleukin (IL)-6, IL-8, IL-1β, and tumor necrosis factor (TNF), which promote various inflammatory responses that cause the dilation of blood vessels, promoting vascular permeability or leading to fever, pain, tissue damage, severe organ necrosis, and even death. Therefore, compounds that reduce the release of EV-induced cytokines are thought to alleviate inflammation (Table 4).

**TABLE 4:** EV-stimulated inflammation inhibitors.

No.	Name	Structure	Target	Inflammatory cytokines release inhibition (%)					References
					
				IL-6	TNF-α	IL-1α	IL-1β	IL-8	
**20**	Salbutamol	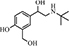	Unknown	46	99	—	—	—	
									Kim et al., 2018

**21**	Nortriptyline	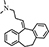	Unknown	48	95	—	—	—	

**22**	Ethyl pyruvate	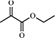	Caspase-11 NLRP3	—	—	95	97	—	Qiu et al., 2020

**23**	NAC	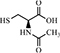	Cysteine utilization	—	73	—	—	—	Volgers et al., 2017a

**24**	Thymol	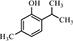	NF-κB MAPKs	50	67	—	67	64	Kwon et al., 2018

**25**	ATRA	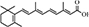	TLR2 IκB NF-κB	62	48	—	—	—	Sinha et al., 2017

**26**	HMW HA	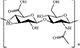	CD44	53	24	—	—	—	Liu et al., 2019

**27**	BUD	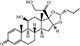	Unknown		83	—	—	—	
									Volgers et al., 2017b

**28**	FLUT	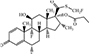	Unknown		84	—	—	—	

**29**	Astragalin	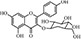	NF-κB	43	—	—	—	55	
									Kou et al., 2008

**30**	MPPG	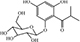	NF-κB	91	—	—	—	95	

**31**	Pep19-2.5	Sequence: GCKKYRRFRWKFKGK FWFWG	Inflammasomes/IL-1 axis	—	—	—	35	—	

**32**	Polymyxin B	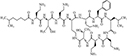	Unknown	—	—	—	75	—	
									Pfalzgraff et al., 2019

**33**	TAK-242	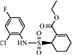	TLR4	—	—	—	65	—	

*— represents no experimental data.*

EVs which are derived from Gram-negative bacteria contain various virulence factors. Moreover, they have been proven to induce systemic inflammatory response syndrome (SIRS) (e.g., leukopenia, thrombocytopenia, increasing the IL-6 and TNF-α concentration or sepsis) (Park et al., 2010). A blockade of IL-6 or TNF-α was confirmed to increase the survival of patients with sepsis (Riedemann et al., 2003). Using novel EV-based screening systems, drugs that showed a dose-dependent suppression of the release of IL-6 and TNF-α from EV-stimulated macrophages and which alleviated EV-induced SIRS were selected. Salbutamol (**20**), a β2 adrenergic receptor agonist, and nortriptyline (**21**) exhibited inhibitory effects in the secretion of *E. coli* EV-stimulated IL-6 and TNF-α both *in vitro* and *in vivo*, and also ameliorated EV-induced SIRS *in vivo*. The release of IL-6 decreased by about 46% and TNF-α was almost undetectable following treatment with 50 μg **20** in EV-induced sublethal SIRS model serum. Treatment with 100 μg **21** resulted in an approximately 48 and 95% reduction in IL-6 and TNF-α in the serum from an EV-induced sublethal SIRS model. As the concentration increased, the inhibitory effect on inflammatory cytokines also increased. In addition, when compared with the results acquired following treatment with **20** alone, the combination of **20** with **21** exhibited an improved inhibitory effect on the secretion of IL-6 and TNF-α (Kim et al., 2018). These findings may help to develop drugs for synergistic therapies for the treatment of systemic inflammatory diseases, including EV-mediated sepsis.

**FIGURE 3:**
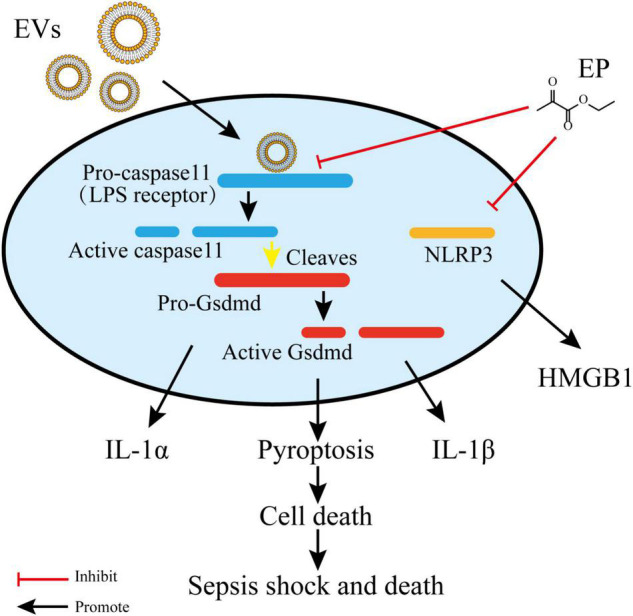
Ethyl pyruvate inhibits caspase-11 expression and NLRP3 activation, which eventually retards pyroptosis and pro-inflammatory responses. Gram-negative EVs carry LPS which are recognized by the receptor and activated caspase-11. Activated caspase-11 then cleaved Gasdermin D (Gsdmd), which produced active Gsdmd to assist in the release of IL-1α and IL-1β, thus triggering cellular pyroptosis. Ethyl pyruvate can suppress LPS binding to caspase-11 and decrease the LPS-induced upregulation of caspase-11. In addition, Ethyl pyruvate prevent the activation of NLRP3 inflammasome which resulted in the release of HMGB1.

LPS is a critical virulence factor of the Gram-negative bacterial outer cell membrane, which can be carried by EVs (Nakao et al., 2014). Ethyl pyruvate (**22**) is a simple aliphatic ester that originates from endogenous metabolites and has been found to have a protective effect on endotoxemia and experimental sepsis (Su et al., 2011). Qiu et al. (2020) demonstrated that **22** treatment could significantly reduce caspase-11 and gasdermin D-mediated pyroptosis stimulated by cytoplasmic LPS and Gram-negative bacterial EVs. Both LPS- and EV-induced IL-1α and IL-1β release was significantly inhibited by **22** treatment in a concentration-dependent manner. Compared to the *E. coli* EVs-stimulated group, treatment with 15 mM **22** led to a reduction of approximately 95% in the level of IL-1α and a reduction of approximately 97% in the level of IL-1β. Moreover, bacterial EVs were automatically integrated into cytoplasm, and the LPS of EVs interacted with and activated caspase-11. Activated caspase-11 then cleaved Gasdermin D (Gsdmd), which produced active Gsdmd to assist in the release of IL-1α and IL-1β, thus triggering cellular pyroptosis. Treatment with **22** can suppress LPS binding to caspase-11 and decrease the LPS-induced upregulation of caspase-11. In addition, **22** treatment dose-dependently prevented the activation of the Pyrin domain containing protein 3 (NLRP3) inflammasome, a multiprotein complex which activated caspase-1 and resulted in the release of high mobility group protein B1 (HMGB1). These findings imply that **22** treatment suppresses multiple signals in the immune responses stimulated by LPS or EVs (Figure 3; Qiu et al., 2020).

N-acetyl-L-cysteine (NAC) (**23**) is a mucolytic agent that is commonly administered with thiol and functions by breaking the disulphide bonds in mucus (Poole et al., 2019). Several studies have suggested that **23** also has various bacteriostatic properties (Zhao and Liu, 2010). Volgers et al. (2017a) investigated whether **23** treatment also affected bacterial EVs. The results revealed that the pro-inflammatory responses induced by bacterial EVs were affected by **23** treatment. In addition, 25 mM **23** reduced TNF-α release by naïve macrophages by approximately 73, 62, and 57% following stimulation with *H. influenzae*, *M. catarrhalis*, and *P. aeruginosa* EVs, respectively. The response of macrophages to bacterial EVs was dose-dependently decreased in the presence of **23**; however, the addition of **23** led to an increase in the secretion of bacterial EVs. This finding verified that **23** was able to counteract the increased EV release and associated pro-inflammatory effects by inhibiting the secretion of pro-inflammatory cytokines. Therefore, **23** exerted a bacteriostatic effect even though it promoted the production of pro-inflammatory EVs. The proposed mechanism through which **23** mediates its bacteriostatic effect might be via the inhibition of cysteine utilization, which can also explain the species-differences in the effects of **23** since the cysteine requirements vary between strains (Volgers et al., 2017a).

**FIGURE 4:**
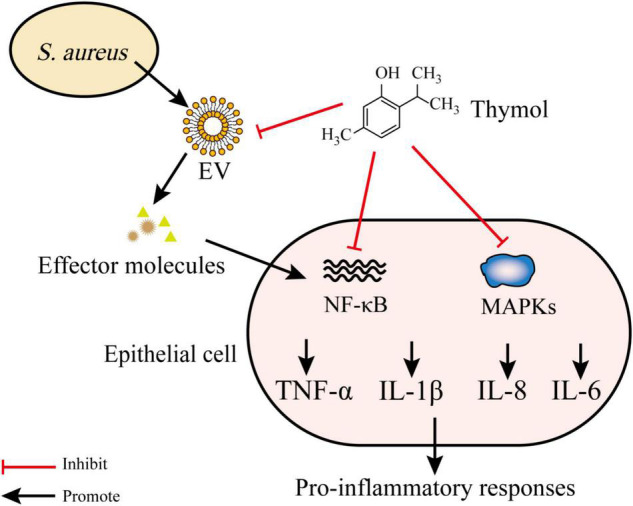
Thymol disrupts EVs and inhibits NF-κB and MAPKs pathways. *S. aureus* EVs contain various effector molecules which trigger NF-κB and MAPKs and lead to the release of proinflammatory cytokines. Thymol reduce proinflammatory cytokines production by inhibiting effector molecules transfer to host and inhibiting NF-κB and MAPKs pathways.

Thymol (2-isopropyl-5-methylphenol) (**24**) is a phenolic monoterpene that is known to possess various pharmacological processes (e.g., anti-inflammatory and antibacterial processes) (Li et al., 2017). Sub-inhibitory concentrations of **24** inhibited the release of toxins [e.g., α-hemolysin (Hla)] by *S. aureus*. Furthermore, the addition of **24** disrupted *S. aureus* EVs and reduced the expression of pro-inflammatory cytokines induced by EVs. Compared to the *S. aureus* EV-treated group, the gene expression of IL-1β, IL-6, IL-8, and TNF-α were decreased by 67, 50, 64, and 67% following treatment with 5 μg **24**, respectively. The topical application of **24** impairs the worsening of atopic dermatitis induced by *S. aureus* EVs in an *in vivo* model. Damage of bacterial EVs by treatment with **24** suppressed the delivery of the effector molecules to host, which led to the lower stimulation of pro-inflammatory responses. Moreover, **24** decreased the production of pro-inflammatory cytokines through the suppression of nuclear factor-κB (NF-κB) and mitogen-activated protein kinases (MAPKs). Thus, it is possible that inflammatory responses caused by thymol-treated *S. aureus* EVs are inhibited by both the suppression of the transmission of effector molecules (e.g., peptidoglycans) to the host and the inflammatory signaling pathways related to inflammation (Figure 4; Kwon et al., 2018, 2019).

Bacterial EVs are considered to represent as novel vaccine candidates due to their excellent biocompatibility. However, the toxicity associated with inducing pro-inflammatory responses represents a major concern in EV-related vaccines (Lee et al., 2017; Fingermann et al., 2018). Hence, all-trans retinoic acid (ATRA) (**25**) was selected to suppress EV-induced toxicity. The study by Sinha et al. (2017) demonstrated that pretreatment with ATRA inhibited *V. cholerae* EV-induced inflammation without suppressing the protective immune response. In a mouse model, **25** treatment decreased *V. cholerae* EV-induced pro-inflammatory cytokines. Compared to the mice administered EVs alone, the level of IL-6 and TNF-α was decreased by approximately 64 and 48% when treated with *cholera* pentavalent EVs in conjunction with 37.5 mg/mL **25** for 3 days. Furthermore, treatment with 1 mM **25** for 16 h reduced *V. cholerae* EV-mediated TLR2 expression. **25** treatment also inhibited the downstream signaling of CPMVs (e.g., degradation of IκB and nuclear translocation of NF-κB), which contributed to the reduction of pro-inflammatory cytokines (Sinha et al., 2017).

Hyaluronic acid (HA) is key factor involved in maintaining the structure and homeostasis of the alveolar air-blood barrier. HA can be degraded to a low-molecular-weight and led to inflammatory responses (Jiang et al., 2010; Lennon and Singleton, 2011; Liang et al., 2016). Surprisingly, high molecular weight hyaluronic acid (HMW HA) (**26**) had the opposite effects. Liu et al. (2019) studied the effects of **26** on the lung injury induced by *E. coli* secreted EVs. Under the co-existence of *E. coli* EVs and 20 μg/mL **26**, the release of TNF-α and IL-6 by human monocytes was reduced by 24 and 53%, respectively. CD44 is a transmembrane glycoprotein typically expressed on immune cell EVs. In this study, it was discovered that the binding of **26** with *E. coli* EVs, which was partly dependent on CD44, prevented EV uptake by human monocytes and reduced *E. coli* EV-induced inflammatory responses. Unexpectedly, **26** administration reduced the total colony forming units (CFUs) of bacteria. Possible explanations for its antimicrobial effect may be the activation of CD44, which is involved in phagocytosis and interference with the adhesion of bacteria to a cellular substrate (Liu et al., 2019).

Recent studies show that hormones (e.g., steroid hormones) could directly interfere with bacterial growth, virulence, and gene expression (Freestone, 2013; Earl et al., 2015). Whether the treatment of glucocorticoids, budesonide (BUD) (**27**) and fluticasone (FLUT) (**28**), affected bacterial EV secretion and suppressed EVs induced pro-inflammatory responses was investigated. For several bacterial species, including *H. influenzae*, *P. aeruginosa*, *S. pneumoniae*, and *M. catarrhiae*, the release of TNF-α and the pro-inflammatory effects stimulated by pathogenic EVs were decreased following treatment with **27** and **28**. Treatment of 0.1 μM **27** and **28** decreased TNF-α secretion by macrophages by approximately 84 and 83%, respectively in response to *M. catarrhiae* EVs. However, neither the release of EVs nor bacterial growth were affected (Volgers et al., 2017b). These findings suggest that **27** and **28** may have a positive effect on bacterial EV-induced inflammation, despite the observation that EV release and bacterial growth remained unaltered.

Vesicles secreted from *P. gingivalis*, a crucial etiologic agent in chronic marginal periodontitis, were considered to be more virulent weapons than bacterial cells because they could destruct the periodontal tissues and stimulate host cells to activate various inflammatory responses (Inaba et al., 2005). There was evidence demonstrated that hop bract polyphenol (HBP) inhibited the expression of cyclooxygenase-2 (COX-2), IL-6, and -8, matrix metalloproteinases (MMP)-1 and -3 in a dose-dependent manner. Further fractionation of HBP identified that the effective components were astragalin (**29**) and 2-[(2-methylpropanoyl)-phloroglucinol]1-O-b-D-glucopyranoside (MPPG) (**30**). Compared to human gingival epithelial cells (HGE cells) treated with vesicles in the absence of HBP, the expression of COX-2, IL-6, IL-8, MMP-1, and MMP-3 were all significantly decreased in the presence of HBP. Further investigation suggested that 10 μg/mL **30** blocked over 90% of the expression of these pro-inflammatory mRNAs. Treatment with 25 μg/mL **29** also significantly suppressed the expression of the inflammatory mediators. These results implied that the anti-inflammatory efficacy of HBP contributed to **29** and **30**. The effects of **29** and **30** may be due to the inhibition of HGE cells on NF-κB activation and the proteolytic activities of *P. gingivalis*. In addition, safety experiments showed that HBP, **29,** and **30** showed low cytotoxicity (Kou et al., 2008). Therefore, HBP, **29** and **30** are suggested to have potential for the treatment of periodontitis.

Pfalzgraff et al. (2016, 2017) and Heinbockel et al. (2018) previously demonstrated that one type of synthetic antiendotoxin peptide, Pep19-2.5 (**31**), could efficiently neutralize the inflammatory responses mediated by extracellular and intracellular LPS. Furthermore, the authors also found that treatment with **31** and polymyxin B (**32**) decreased *E. coli* EV-stimulated IL-1β and TNF secretion in macrophages and suppressed *E. coli* EV-mediated pyroptosis. Treatment with 10 μg/mL **31** and **32** reduced IL-1β release by THP-1 macrophages by about 35 and 75%, respectively. Moreover, the TLR4 signaling inhibitor, TAK-242 (**33**), also inhibited EV-induced IL-1β and TNF release, but not reduce pyroptosis. Treatment with 1 μg/mL **33** reduced IL-1β release from THP-1 macrophages by about 65%. The results of the Limulus assay showed that the reduction of these inflammatory signals resulted from a blockade of LPS-induced stimulation. It was also demonstrated that Pep19-2.5-neutralized EV release of LPS. After the internalization of EVs and Pep19-2.5, Pep19-2.5 may suppress the binding of EV-released LPS to inflammatory caspases. Other evidence has shown that **31** suppressed the EV-mediated activation of the inflammasome/IL-1 axis in macrophages, which may be related to protection against sepsis (Pfalzgraff et al., 2019).

### Inhibitors That Reduce the Activity of Extracellular Vesicles

Due to the multiple physiological properties of bacterial EVs in host cells, compounds that destroy their structure or inhibit the activity of their substances also affect their functionality (Table 5).

**TABLE 5:** Inhibitors that reduce the activity of EVs.

No.	Name	Structure	Target	Activity	References
**34**	EGCg	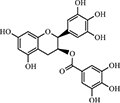	LtxA	MIC = 10 μg/mL	Chang et al., 2020

*Aggregatibacter actinomycetemcomitans (A. actinomycetemcomitans)* is an oral pathogen that produces EVs which deliver various virulence factors, including leukotoxin A (LtxA), a leukotoxin that can specifically kill human white blood cells. Interference with LtxA activity showed a possible approach to decreasing bacterial pathogenicity. Previous studies have shown that galloylated catechins can block the delivery of LtxA to the host by altering the secondary structure of the toxin and inhibiting its binding to cholesterol in the host cell membrane. Epigallocatechin gallate (EGCg) (**34**), a galloylated catechin, can inhibit *A. actinomycetemcomitans* growth at micromolar concentrations. The minimal inhibitory concentration (MIC) of **34** to *A. actinomycetemcomitans* was 10 μg/mL and the inhibitory effect was further increased with the increase in the concentration of **34**. At subinhibitory concentrations, **34** increased LtxA production; however, the level of bacterial toxicity toward immune cells was decreased. Therefore, these contradictory results were further investigated. When *A. actinomycetemcomitans* EVs were used as a model of the bacterial membrane, treatment with **34** enhanced the binding of LtxA to EVs, which might affect the surface components and alter the offensive function of the EVs. It is also conceivable that **34** promoted the association of LtxA with the bacterial membranes. The promoting effect of **34** on the binding of LtxA and bacterial membranes and EVs may be a reason for such reduced immunotoxicity (Chang et al., 2020).

Except for their effect of inhibiting bacterial EV production, both **18** and **19**, could also inhibit the activity of EVs. In addition, **18** decreased the hemolytic activity of *S. aureus* EVs, which was dependent on the inhibition of hemolysin (e.g., α-toxin) in EVs. **18** might also destroy the integrity of EVs, thus preventing the delivery of hemolysin to the host (Mitsuwan et al., 2019). Treatment with **19** decreased the staphylococcal EV-induced myeloperoxidase (MPO) activity and vascular permeability, as well as Hla (An et al., 2019).

### Other Inhibitors

Vascular endothelial growth factor (VEGF) has been shown to be an effective inducer of vascular permeability and angiogenesis (Apte et al., 2019; Guo et al., 2021). Studies have shown enhanced VEGF expression in human gingival fibroblasts (HGFs) stimulated with the major etiologic agents of periodontitis (Ramya and Kumar, 2014). Nunez et al. (2010) confirmed that resveratrol (**35**) significantly suppressed VEGF production by HGF in response to periodontopathic bacterial vesicles and outer membrane proteins. Moreover, **35** treatment reduced the vascular permeability induced by bacterial vesicles and outer membrane proteins. Vesicle-stimulated VEGF production was reduced by approximately 75 and 60% in *A. actinomycetemcomitans* and *P. gingivalis* under 80 μM **35**, respectively (Table 6). The MAPK signaling pathway was identified as one of the targets of **35** in endothelial cells. The inhibitory efficacy of **35** was also related to the suppression of proteases (e.g., MMPs, a type of proangiogenic factor). Notably, the antioxidative activity of **35** was associated with anti-angiogenesis. A decrease in oxidative stress by **35** resulted in the inhibition of transcription factors, including protein-1 and NF-κB, which modulated the expression of VEGF. In contrast to other angiogenic factor inhibitors, **35** has the advantage of blocking the common angiogenic pathway, which was activated by different angiogenic factors. Furthermore, **35** can be administered orally (Nunez et al., 2010).

**TABLE 6:** Other inhibitors.

No.	Name	Structure	Target	Activity	References
**35**	Resveratrol	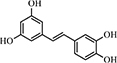	MAPKs protein-1 NF-κB	Reduce about 75% and 60% VEGF production in *A. actinomycetemcomitans* and *P. gingivalis*	Nunez et al., 2010

In summary, bacterial EV inhibitors is likely an effective approach to bacterial infections. Both chemical drugs and multiple natural products also displayed inhibitory activities, which acted on the entire process of bacterial EV production, including their formation, transport, uptake by host cells, and pro-inflammatory activity. In addition to being used alone, these inhibitors could also be used as adjuncts to antibiotics or other antimicrobial agents, thereby enhancing antimicrobial activity or reducing bacterial resistance.

## Conclusion

The secretion of EVs is an important method by which bacteria exert their invasive power. EVs typically mediate bacterial interactions with a host or other symbiotic bacteria. The development of drugs targeting bacterial EVs has the potential to provide new insight into the intractable problems associated with bacterial infections.

Recently, inhibitors targeting bacterial EVs have developed significantly. Several commercial drugs have been demonstrated to be effective at inhibiting bacterial EVs, which will greatly aid in the discovery of new compounds and the development of new uses for old drugs; however, there are still some limitations. Although the aforementioned compounds exhibit substantial inhibitory activity against bacterial EVs, the precise mechanisms of many compounds remain unclear. For example, while PQS is related to EV production, the method by which it affects EV production and how inhibitors suppress EV formation by inhibiting PQS remains to be explored. In addition to inhibiting the production of EVs and the EV-stimulated inflammatory response, other directions, including the prevention of their induced immune escape and antibiotic resistance are also worthy of further study. The systematic classification of inhibitors and structure-activity relationships should also be further investigated.

Bacterial EVs also exhibit numerous special properties (e.g., great integrity and stability, immunogenicity, protection of inclusions, ability to be mass-produced, and ease of operation). Due to these effects, EVs have the potential to be used in vaccine development and as drug carriers. For example, Shiga toxin-producing *E. coli* (STEC) EVs can be developed into a vaccine against Hemolytic Uremic Syndrome (HUS) in mice (Fingermann et al., 2018). Moreover, EVs from *N. meningitides*, which has been used as a vaccine for preventing meningococcal disease, has acquired licenses in many countries (Martinon-Torres et al., 2017). A modified EV with low endotoxicity as a vaccine adjuvant of an influenza intranasal vaccine has shown higher protection and lower toxicity after the co-administration compared to the vaccine-alone administration (Lee et al., 2017). EVs can also be engineered to target a particular region of a host; thereby functioning as a targeted drug carrier (Bitto and Kaparakis-Liaskos, 2017). Recent studies have focused on the use of bacterial EVs in oncology therapies. Bacterial EVs have a remarkable ability to stimulate an anticancer immune response and to eradicate established tumors (Kim et al., 2017). Although substantial progress has been made in the application of bacterial EVs and EV-associated compounds in biomedicine, numerous concerns still remain. The inhibitory activity, specificity, and toxic side effects of EVs still require further study. Moreover, challenges associated with methods of mass production need to be resolved for clinical applications. Thus, modified and artificial bacterial EVs represent a direction worthy of further investigation. Therefore, overcoming these challenges should be considered in future studies.

Overall, the natural properties of bacterial EVs exhibit double-edged effects in a host. As we learn more about the mechanisms of bacterial EVs, they will be of great benefit in the development of new agents or therapies to intractable diseases. Although EVs have not been studied in depth enough, these early studies provide valuable knowledge for the development of effective, highly selective, and well-tolerated therapeutics.

## Author Contributions

All authors listed have made a substantial, direct, and intellectual contribution to the work, and approved it for publication.

## Conflict of Interest

The authors declare that the research was conducted in the absence of any commercial or financial relationships that could be construed as a potential conflict of interest.

## Publisher’s Note

All claims expressed in this article are solely those of the authors and do not necessarily represent those of their affiliated organizations, or those of the publisher, the editors and the reviewers. Any product that may be evaluated in this article, or claim that may be made by its manufacturer, is not guaranteed or endorsed by the publisher.

## Abbreviations

4HI: 4-hydroxyindole
LtxA: Leukotoxin A
5HI: 5-hydroxyindole
MAPKs: Mitogen-activated protein kinases
6HI: 6-hydroxyindole
MHC-II: Major histocompatibility complex-II
7HI: 7-hydroxyindole
MIC: Minimal inhibitory concentration
Ag85: Antigen Ag85 complex
MMP: Matrix metalloproteinases
ATRA: All-trans Retinoic Acid
MPO: Myeloperoxidase
BUD: Budesonide
MRSA: Methicillin-resistant *S. aureus*
CBD: Cannabidiol
MVs: Membrane vesicles
CFUS: Colony forming units
COX-2: Cyclooxygenase-2
NAC: N-acetyl -L-cysteine
DCs: Dendritic cells
NETs: Neutrophil extracellular traps
EGCg: Epigallocatechin gallate
NF- κ B: Nuclear factor- κ B
EVs: Extracellular vesicles
NK cells: Natural killing cells
FLUT: Fluticasone
NLRC4: NOD-like receptor family caspase activating and recruitment domain-containing protein 4
FOM: Fosfomycin
NLRP3: Pyrin domain containing protein 3
Gsdmd: Gasdermin D
OMVs: Outer membrane vesicles
HA: Hyaluronic acid
OXA: Oxacillinase
HBHA: Heparin binding hemagglutinin adhesion
PADs: Peptidyl arginine deiminases
HBP: Hop bract polyphenol
PQS: 2-heptyl-3-hydroxy-4-quinolone
HGE cells: Human gingival epithelial cells
QS: Quorum-sensing
HGF: Human gingival fibroblast
SigB: Sigma factor B
Hla: α-Hemolysin
SIRS: Systemic inflammatory response syndrome
HMGB1: High mobility group protein B1
STEC: Shiga toxin-producing Escherichia coli
HUS: Hemolytic Uremic Syndrome
TLR: Toll-like receptor
IL: Interleukin
TNF: Tumor necrosis factor
LPS: Lipopolysaccharides
VEGFs: Vascular endothelial growth factors.

## References

[B1] Ahmadi Badi S. Bruno S. P. Moshiri A. Tarashi S. Siadat S. D. Masotti A. (2020). Small RNAs in outer membrane vesicles and their function in host-microbe interactions. *Front. Microbiol.* 11:1209. 10.3389/fmicb.2020.01209 32670219 PMC7327240

[B2] An Y. Wang Y. Zhan J. Tang X. Shen K. Shen F. (2019). Fosfomycin protects mice from *Staphylococcus aureus* pneumonia caused by alpha-hemolysin in extracellular vesicles by inhibiting MAPK-regulated NLRP3 inflammasomes. *Front. Cell. Infect. Microbiol.* 9:253. 10.3389/fcimb.2019.00253 31380296 PMC6644418

[B3] Apte R. S. Chen D. S. Ferrara N. (2019). VEGF in signaling and disease: beyond discovery and development. *Cell* 176 1248–1264. 10.1016/j.cell.2019.01.021 30849371 PMC6410740

[B4] Athman J. J. Sande O. J. Groft S. G. Reba S. M. Nagy N. Wearsch P. A. (2017). Mycobacterium tuberculosis membrane vesicles inhibit T cell activation. *J. Immunol.* 198 2028–2037. 10.4049/jimmunol.1601199 28122965 PMC5322216

[B5] Avila-Calderon E. D. Araiza-Villanueva M. G. Cancino-Diaz J. C. Lopez-Villegas E. O. Sriranganathan N. Boyle S. M. (2015). Roles of bacterial membrane vesicles. *Arch. Microbiol.* 197 1–10. 10.1007/s00203-014-1042-1047 25294190

[B6] Berleman J. E. Allen S. Danielewicz M. A. Remis J. P. Gorur A. Cunha J. (2014). The lethal cargo of Myxococcus xanthus outer membrane vesicles. *Front. Microbiol.* 5:474. 10.3389/fmicb.2014.00474 25250022 PMC4158809

[B7] Bitto N. J. Kaparakis-Liaskos M. (2017). The therapeutic benefit of bacterial membrane vesicles. *Int. J. Mol. Sci.* 18:1287. 10.3390/ijms18061287 28621731 PMC5486109

[B8] Blaskovich M. A. T. Kavanagh A. M. Elliott A. G. Zhang B. Ramu S. Amado M. (2021). The antimicrobial potential of cannabidiol. *Commun. Biol.* 4:7. 10.1038/s42003-020-01530-y 33469147 PMC7815910

[B9] Briaud P. Carroll R. K. (2020). Extracellular vesicle biogenesis and functions in gram-positive bacteria. *Infect. Immun.* 88:e00433-20. 10.1128/IAI.00433-420 PMC7671900 32989035

[B10] Brown L. Wolf J. M. Prados-Rosales R. Casadevall A. (2015). Through the wall: extracellular vesicles in gram-positive bacteria, mycobacteria and fungi. *Nat. Rev. Microbiol.* 13 620–630. 10.1038/nrmicro3480 26324094 PMC4860279

[B11] Chang E. H. Giaquinto P. Huang J. Balashova N. V. Brown A. C. (2020). Epigallocatechin gallate inhibits leukotoxin release by *Aggregatibacter actinomycetemcomitans* by promoting association with the bacterial membrane. *Mol. Oral. Microbiol.* 35 29–39. 10.1111/omi.12275 31816197 PMC7015128

[B12] Chen J. W. Guo Y. Q. Lu Y. J. Wang B. X. Sun J. D. Zhang H. W. (2019). Chemistry and biology of siderophores from marine microbes. *Mar. Drugs* 17:562. 10.3390/md17100562 31569555 PMC6836290

[B13] Chen J. W. Lu Y. J. Ye X. Y. Emam M. Zhang H. W. Wang H. (2020). Current advances in *Vibrio harveyi* quorum sensing as drug discovery targets. *Eur. J. Med. Chem.* 207:112741. 10.1016/j.ejmech.2020.112741 32871343

[B14] Chen J. W. Wu Q. H. Hua Y. Chen J. Zhang H. W. Wang H. (2017). Potential applications of biosurfactant rhamnolipids in agriculture and biomedicine. *Appl. Microbiol. Biot.* 101 8309–8319. 10.1007/s00253-017-8554-8554 29018916

[B15] Cheng Y. Schorey J. S. (2019). Extracellular vesicles deliver *Mycobacterium* RNA to promote host immunity and bacterial killing. *EMBO Rep.* 20:e46613. 10.15252/embr.201846613 30683680 PMC6399609

[B16] Choi J. W. Kim S. C. Hong S. H. Lee H. J. (2017). Secretable small RNAs via outer membrane vesicles in periodontal pathogens. *J. Dent. Res.* 96 458–466. 10.1177/0022034516685071 28068479

[B17] Codemo M. Muschiol S. Iovino F. Nannapaneni P. Plant L. Wai S. N. (2018). Immunomodulatory effects of pneumococcal extracellular vesicles on cellular and humoral host defenses. *mBio* 9:15. 10.1128/mBio.00559-518 PMC5893880 29636428

[B18] Depke M. Burian M. Schafer T. Broker B. M. Ohlsen K. Volker U. (2012). The alternative sigma factor B modulates virulence gene expression in a murine *Staphylococcus aureus* infection model but does not influence kidney gene expression pattern of the host. *Int. J. Med. Microbiol.* 302 33–39. 10.1016/j.ijmm.2011.09.013 22019488

[B19] Diggle S. P. Winzer K. Chhabra S. R. Worrall K. E. Camara M. Williams P. (2003). The *Pseudomonas aeruginosa* quinolone signal molecule overcomes the cell density-dependency of the quorum sensing hierarchy, regulates rhl-dependent genes at the onset of stationary phase and can be produced in the absence of LasR. *Mol. Microbiol.* 50 29–43. 10.1046/j.1365-2958.2003.03672.x 14507361

[B20] Ding F. X. Liu B. Zou W. J. Li Q. B. Tian D. Y. Fu Z. (2018). Pseudomonas aeruginosa-derived exosomes ameliorates allergic reactions via inducing the Treg response in asthma. *Pediatr. Res.* 84 125–133. 10.1038/s41390-018-0020-21 29795208

[B21] Earl C. S. Keong T. W. An S. Q. Murdoch S. McCarthy Y. Garmendia J. (2015). Haemophilus influenzae responds to glucocorticoids used in asthma therapy by modulation of biofilm formation and antibiotic resistance. *EMBO Mol. Med.* 7 1018–1033. 10.15252/emmm.201505088 25995336 PMC4551341

[B22] Ellis T. N. Kuehn M. J. (2010). Virulence and immunomodulatory roles of bacterial outer membrane vesicles. *Microbiol. Mol. Biol. Rev.* 74 81–94. 10.1128/MMBR.00031-39 20197500 PMC2832350

[B23] Fingermann M. Avila L. De Marco M. B. Vazquez L. Di Biase D. N. Muller A. V. (2018). OMV-based vaccine formulations against Shiga toxin producing Escherichia coli strains are both protective in mice and immunogenic in calves. *Hum. Vaccin. Immunother.* 14 2208–2213. 10.1080/21645515.2018.1490381 29923791 PMC6183318

[B24] Freestone P. (2013). Communication between bacteria and their hosts. *Scientifica (Cairo)* 2013:361073. 10.1155/2013/361073 24381789 PMC3871906

[B25] Guo X. Yi H. Li T. C. Wang Y. Wang H. Chen X. (2021). Role of vascular endothelial growth factor (VEGF) in human embryo implantation: clinical implications. *Biomolecules* 11:253. 10.3390/biom11020253 33578823 PMC7916576

[B26] Gurung M. Moon D. C. Choi C. W. Lee J. H. Bae Y. C. Kim J. (2011). Staphylococcus aureus produces membrane-derived vesicles that induce host cell death. *PLoS One* 6:e27958. 10.1371/journal.pone.0027958 22114730 PMC3218073

[B27] Heinbockel L. Weindl G. Martinez-de-Tejada G. Correa W. Sanchez-Gomez S. Barcena-Varela S. (2018). Inhibition of lipopolysaccharide- and lipoprotein-induced inflammation by antitoxin peptide Pep19-2.5. *Front. Immunol.* 9:1704. 10.3389/fimmu.2018.01704 30093904 PMC6070603

[B28] Hernández-Cervantes R. Méndez-Díaz M. Prospéro-García Ó Morales-Montor J. (2017). Immunoregulatory role of cannabinoids during infectious disease. *Neuroimmunomodulation* 24 183–199. 10.1159/000481824 29151103

[B29] Hoekstra D. van der Laan J. W. de Leij L. Witholt B. (1976). Release of outer membrane fragments from normally growing Escherichia coli. *BBA- Biomembranes* 455 889–899. 10.1016/0005-2736(76)90058-90054 793634

[B30] Inaba H. Tagashira M. Kanda T. Ohno T. Kawai S. Amano A. (2005). Apple- and hop-polyphenols protect periodontal ligament cells stimulated with enamel matrix derivative from *Porphyromonas gingivalis*. *J. Periodontol.* 76 2223–2229. 10.1902/jop.2005.76.12.2223 16332233

[B31] Jan A. T. (2017). Outer membrane vesicles (OMVs) of gram-negative bacteria: a perspective update. *Front. Microbiol.* 8:1053. 10.3389/fmicb.2017.01053 28649237 PMC5465292

[B32] Jarzab M. Posselt G. Meisner-Kober N. Wessler S. (2020). Helicobacter pylori-Derived outer membrane vesicles (OMVs): role in bacterial pathogenesis? *Microorganisms* 8:1328. 10.3390/microorganisms8091328 32878302 PMC7564109

[B33] Jhelum H. Sori H. Sehgal D. (2018). A novel extracellular vesicle-associated endodeoxyribonuclease helps *Streptococcus pneumoniae* evade neutrophil extracellular traps and is required for full virulence. *Sci. Rep.* 8:7985. 10.1038/s41598-018-25865-z 29789571 PMC5964101

[B34] Jiang D. Liang J. Noble P. W. (2010). Regulation of non-infectious lung injury, inflammation, and repair by the extracellular matrix glycosaminoglycan hyaluronan. *Anat. Rec. (Hoboken)* 293 982–985. 10.1002/ar.21102 20186964 PMC2877145

[B35] Jurkoshek K. S. Wang Y. Athman J. J. Barton M. R. Wearsch P. A. (2016). Interspecies communication between pathogens and immune cells via bacterial membrane vesicles. *Front. Cell. Dev. Biol.* 4:125. 10.3389/fcell.2016.00125 27891500 PMC5104960

[B36] Kaparakis-Liaskos M. Ferrero R. L. (2015). Immune modulation by bacterial outer membrane vesicles. *Nat. Rev. Immunol.* 15 375–387. 10.1038/nri3837 25976515

[B37] Kim D. K. Kang B. Kim O. Y. Choi D. S. Lee J. Kim S. R. (2013). EVpedia: an integrated database of high-throughput data for systemic analyses of extracellular vesicles. *J. Extracell Vesicles* 2. 10.3402/jev.v2i0.20384 24009897 PMC3760654

[B38] Kim J. H. Lee J. Park K. S. Hong S. W. Gho Y. S. (2018). Drug repositioning to alleviate systemic inflammatory response syndrome caused by gram-negative bacterial outer membrane vesicles. *Adv. Healthc. Mater.* 7:1701476. 10.1002/adhm.201701476 29683274

[B39] Kim O. Y. Park H. T. Dinh N. T. H. Choi S. J. Lee J. Kim J. H. (2017). Bacterial outer membrane vesicles suppress tumor by interferon-gamma-mediated antitumor response. *Nat. Commun.* 8:626. 10.1038/s41467-017-00729-728 PMC5606984 28931823

[B40] Kosgodage U. S. Matewele P. Awamaria B. Kraev I. Warde P. Mastroianni G. (2019a). Cannabidiol is a novel modulator of bacterial membrane vesicles. *Front. Cell. Infect. Microbiol.* 9:324. 10.3389/fcimb.2019.00324 31552202 PMC6747004

[B41] Kosgodage U. S. Matewele P. Mastroianni G. Kraev I. Brotherton D. Awamaria B. (2019b). Peptidylarginine deiminase inhibitors reduce bacterial membrane vesicle release and sensitize bacteria to antibiotic treatment. *Front. Cell. Infect. Microbiol.* 9:227. 10.3389/fcimb.2019.00227 31316918 PMC6610471

[B42] Kosgodage U. S. Mould R. Henley A. B. Nunn A. V. Guy G. W. Thomas E. L. (2018). Cannabidiol (CBD) is a novel inhibitor for exosome and microvesicle (EMV) release in cancer. *Front. Pharmacol.* 9:889. 10.3389/fphar.2018.00889 30150937 PMC6099119

[B43] Kosgodage U. S. Trindade R. P. Thompson P. R. Inal J. M. Lange S. (2017). Chloramidine/bisindolylmaleimide-I-mediated inhibition of exosome and microvesicle release and enhanced efficacy of cancer chemotherapy. *Int. J. Mol. Sci.* 18:1007. 10.3390/ijms18051007 28486412 PMC5454920

[B44] Kou Y. Inaba H. Kato T. Tagashira M. Honma D. Kanda T. (2008). Inflammatory responses of gingival epithelial cells stimulated with Porphyromonas gingivalis vesicles are inhibited by hop-associated polyphenols. *J. Periodontol.* 79 174–180. 10.1902/jop.2008.070364 18166108

[B45] Kovalchuk O. Kovalchuk I. (2020). Cannabinoids as anticancer therapeutic agents. *Cell Cycle* 19 961–989. 10.1080/15384101.2020.1742952 32249682 PMC7217364

[B46] Kulkarni H. M. Nagaraj R. Jagannadham M. V. (2015). Protective role of E. coli outer membrane vesicles against antibiotics. *Microbiol. Res.* 181 1–7. 10.1016/j.micres.2015.07.008 26640046

[B47] Kwon H. I. Jeong N. H. Jun S. H. Son J. H. Kim S. Jeon H. (2018). Thymol attenuates the worsening of atopic dermatitis induced by *Staphylococcus aureus* membrane vesicles. *Int. Immunopharmacol.* 59 301–309. 10.1016/j.intimp.2018.04.027 29679854

[B48] Kwon H. I. Jeong N. H. Kim S. Y. Kim M. H. Son J. H. Jun S. H. (2019). Inhibitory effects of thymol on the cytotoxicity and inflammatory responses induced by *Staphylococcus aureus* extracellular vesicles in cultured keratinocytes. *Microb. Pathogenesis* 134:103603. 10.1016/j.micpath.2019.103603 31226290

[B49] Lee E. Y. Choi D. Y. Kim D. K. Kim J. W. Park J. O. Kim S. (2009a). Gram-positive bacteria produce membrane vesicles: proteomics-based characterization of *Staphylococcus aureus*-derived membrane vesicles. *Proteomics* 9 5425–5436. 10.1002/pmic.200900338 19834908

[B50] Lee J. Attila C. Cirillo S. L. Cirillo J. D. Wood T. K. (2009b). Indole and 7-hydroxyindole diminish *Pseudomonas aeruginosa*. *Microb. Biotechnol.* 21 75–90. 10.1111/j.1751-7915.2008.00061.x 21261883 PMC3815423

[B51] Lee J. H. Choi C. W. Lee T. Kim S. I. Lee J. C. Shin J. H. (2013). Transcription factor sigmaB plays an important role in the production of extracellular membrane-derived vesicles in listeria monocytogenes. *PLoS One* 8:e73196. 10.1371/journal.pone.0073196 23977379 PMC3748028

[B52] Lee J. Bansal T. Jayaraman A. Bentley W. E. Wood T. K. (2007). Enterohemorrhagic *Escherichia coli* biofilms are inhibited by 7-hydroxyindole and stimulated by isatin. *Appl. Environ. Microbiol.* 73 4100–4109. 10.1128/AEM.00360-367 17483266 PMC1932762

[B53] Lee J. Kim S. H. Choi D. S. Lee J. S. Kim D. K. Go G. (2015). Proteomic analysis of extracellular vesicles derived from *Mycobacterium tuberculosis*. *Proteomics* 15 3331–3337. 10.1002/pmic.201500037 26201501

[B54] Lee T. Y. Kim C. U. Bae E. H. Seo S. H. Jeong D. G. Yoon S. W. (2017). Outer membrane vesicles harboring modified lipid a moiety augment the efficacy of an influenza vaccine exhibiting reduced endotoxicity in a mouse model. *Vaccine* 35 586–595. 10.1016/j.vaccine.2016.12.025 28024958 PMC7115551

[B55] Leitao J. H. (2020). Microbial virulence factors. *Int. J. Mol. Sci.* 21:5320. 10.3390/ijms21155320 32727013 PMC7432612

[B56] Lennon F. E. Singleton P. A. (2011). Role of hyaluronan and hyaluronan-binding proteins in lung pathobiology. *Am. J. Physiol. Lung Cell. Mol. Physiol.* 301 L137–L147. 10.1152/ajplung.00071.2010 21571904 PMC3154626

[B57] Li Y. Wen J. M. Du C. J. Hu S. M. Chen J. X. Zhang S. G. (2017). Thymol inhibits bladder cancer cell proliferation via inducing cell cycle arrest and apoptosis. *Biochem. Biophys. Res. Commun.* 491 530–536. 10.1016/j.bbrc.2017.04.009 28389245

[B58] Liang J. Jiang D. Noble P. W. (2016). Hyaluronan as a therapeutic target in human diseases. *Adv. Drug Deliv. Rev.* 97 186–203. 10.1016/j.addr.2015.10.017 26541745 PMC4753080

[B59] Liao Y. T. Kuo S. C. Chiang M. H. Lee Y. T. Sung W. C. Chen Y. H. (2015). *Acinetobacter baumannii* extracellular OXA-58 is primarily and selectively released via outer membrane vesicles after sec-dependent periplasmic translocation. *Antimicrob. Agents Chemother.* 59 7346–7354. 10.1128/AAC.01343-1315 26369971 PMC4649246

[B60] Limsuwan S. Trip E. N. Kouwen T. R. Piersma S. Hiranrat A. Mahabusarakam W. (2009). Rhodomyrtone: a new candidate as natural antibacterial drug from *Rhodomyrtus tomentosa*. *Phytomedicine* 16 645–651. 10.1016/j.phymed.2009.01.010 19303274

[B61] Liu A. R. Park J. H. Zhang X. W. Sugita S. Naito Y. Lee J. H. (2019). Therapeutic effects of hyaluronic acid in bacterial pneumonia in ex vivo perfused human lungs. *Am. J. Resp. Crit. Care* 200 1234–1245. 10.1164/rccm.201812-2296OC 31390880 PMC6857490

[B62] Magnadóttir B. Hayes P. Hristova M. Bragason B. T. Nicholas A. P. Dodds A. W. (2018). Post-translational protein deimination in cod (*Gadus morhua* L.) ontogeny novel roles in tissue remodelling and mucosal immune defences? *Dev. Comp. Immunol.* 87 157–170. 10.1016/j.dci.2018.06.006 29908202

[B63] Martinon-Torres F. Safadi M. A. P. Martinez A. C. Marquez P. I. Torres J. C. T. Weckx L. Y. (2017). Reduced schedules of 4CMenB vaccine in infants and catch-up series in children: immunogenicity and safety results from a randomised open-label phase 3b trial. *Vaccine* 35 3548–3557. 10.1016/j.vaccine.2017.05.023 28533054

[B64] Mashburn-Warren L. Howe J. Garidel P. Richter W. Steiniger F. Roessle M. (2008). Interaction of quorum signals with outer membrane lipids: insights into prokaryotic membrane vesicle formation. *Mol. Microbiol.* 69 491–502. 10.1111/j.1365-2958.2008.06302.x 18630345 PMC2615190

[B65] Mitsuwan W. Jimenez-Munguia I. Visutthi M. Sianglum W. Jover A. Barcenilla F. (2019). Rhodomyrtone decreases *Staphylococcus aureus* SigB activity during exponentially growing phase and inhibits haemolytic activity within membrane vesicles. *Microb. Pathogenesis* 128 112–118. 10.1016/j.micpath.2018.12.019 30583020

[B66] Nakao R. Takashiba S. Kosono S. Yoshida M. Watanabe H. Ohnishi M. (2014). Effect of Porphyromonas gingivalis outer membrane vesicles on gingipain-mediated detachment of cultured oral epithelial cells and immune responses. *Microbes Infect.* 16 6–16. 10.1016/j.micinf.2013.10.005 24140554

[B67] Nunez M. J. Novio S. Balboa J. Seoane J. Suarez J. A. Freire-Garabal M. (2010). Effects of resveratrol on expression of vascular endothelial growth factor in human gingival fibroblasts stimulated by periodontal pathogens. *Acta Odontol. Scand.* 68 239–247. 10.3109/00016357.2010.494269 20507262

[B68] Ohno S. Drummen G. P. Kuroda M. (2016). Focus on extracellular vesicles: development of extracellular vesicle-based therapeutic systems. *Int. J. Mol. Sci.* 17:172. 10.3390/ijms17020172 26861303 PMC4783906

[B69] Papayannopoulos V. (2017). Neutrophil extracellular traps in immunity and disease. *Nat. Rev. Immunol.* 18 134–147. 10.1038/nri.2017.105 28990587

[B70] Park K. S. Choi K. H. Kim Y. S. Hong B. S. Kim O. Y. Kim J. H. (2010). Outer membrane vesicles derived from *Escherichia coli* induce systemic inflammatory response syndrome. *PLoS One* 5:e11334. 10.1371/journal.pone.0011334 20596524 PMC2893157

[B71] Peng Y. Yin S. Wang M. (2020). Extracellular vesicles of bacteria as potential targets for immune interventions. *Hum. Vaccin. Immunother*. 17 897–903. 10.1080/21645515.2020.1799667 32873124 PMC7993133

[B72] Pesci E. C. Milbank J. B. J. Pearson J. P. McKnight S. Kende A. S. Greenberg E. P. (1999). Quinolone signaling in the cell-to-cell communication system of *Pseudomonas aeruginosa*. *Proc. Natl. Acad. Sci. U S A.* 96 11229–11234. 10.1073/pnas.96.20.11229 10500159 PMC18016

[B73] Pfalzgraff A. Correa W. Heinbockel L. Schromm A. B. Lubow C. Gisch N. (2019). LPS-neutralizing peptides reduce outer membrane vesicle-induced inflammatory responses. *BBA-Mol. Cell. Biol. Lipids* 1864 1503–1513. 10.1016/j.bbalip.2019.05.018 31163264

[B74] Pfalzgraff A. Heinbockel L. Su Q. Brandenburg K. Weindl G. (2017). Synthetic anti-endotoxin peptides inhibit cytoplasmic LPS-mediated responses. *Biochem. Pharmacol.* 140 64–72. 10.1016/j.bcp.2017.05.015 28539262

[B75] Pfalzgraff A. Heinbockel L. Su Q. Gutsmann T. Brandenburg K. Weindl G. (2016). Synthetic antimicrobial and LPS-neutralising peptides suppress inflammatory and immune responses in skin cells and promote keratinocyte migration. *Sci. Rep.* 6:31577. 10.1038/srep31577 27509895 PMC4980674

[B76] Poeppl W. Tobudic S. Lingscheid T. Plasenzotti R. Kozakowski N. Lagler H. (2011). Daptomycin, fosfomycin, or both for treatment of methicillin-resistant Staphylococcus aureus osteomyelitis in an experimental rat model. *Antimicrob. Agents Chemother.* 55 4999–5003. 10.1128/AAC.00584-511 21859942 PMC3194995

[B77] Poole P. Sathananthan K. Fortescue R. (2019). Mucolytic agents versus placebo for chronic bronchitis or chronic obstructive pulmonary disease. *Cochrane Database Syst. Rev.* 5:CD001287. 10.1002/14651858.CD001287.pub6 31107966 PMC6527426

[B78] Prados-Rosales R. Weinrick B. C. Pique D. G. Jacobs W. R. Casadevall A. Rodriguez G. M. (2014). Role for *Mycobacterium tuberculosis* membrane vesicles in iron acquisition. *J. Bacteriol.* 196 1250–1256. 10.1128/jb.01090-1013 24415729 PMC3957709

[B79] Qiu X. H. Cheng X. Y. Zhang J. Yuan C. Zhao M. Y. Yang X. Y. (2020). Ethyl pyruvate confers protection against endotoxemia and sepsis by inhibiting caspase-11-dependent cell pyroptosis. *Int. Immunopharmacol.* 78:106016. 10.1016/j.intimp.2019.106016 31796383

[B80] Ramya Kumar S. (2014). Expression of VEGF in periodontal tissues of type II diabetes mellitus patients with chronic periodontitis -an immunohistochemical study. *J. Clin. Diagn. Res.* 8 ZC01–ZC03. 10.7860/JCDR/2014/7772.4664 25302255 PMC4190781

[B81] Riedemann N. C. Neff T. A. Guo R. F. Bernacki K. D. Laudes I. J. Sarma J. V. (2003). Protective effects of IL-6 blockade in sepsis are linked to reduced C5a receptor expression. *J. Immunol.* 170 503–507. 10.4049/jimmunol.170.1.503 12496437

[B82] Rivera J. Cordero R. J. B. Nakouzi A. S. Frases S. Nicola A. Casadevall A. (2010). Bacillus anthracis produces membrane-derived vesicles containing biologically active toxins. *Proc. Natl. Acad. Sci. U S A.* 107 19002–19007. 10.1073/pnas.1008843107 20956325 PMC2973860

[B83] Rui L. Reardon K. F. Wood T. K. (2004). Protein engineering of toluene ortho-monooxygenase of *Burkholderia cepacia* G4 for regiospecific hydroxylation of indole to form various indigoid compounds. *Appl. Microbiol. Biot.* 66 422–429. 10.1007/s00253-004-1698-z 15290130

[B84] Rumbo C. Fernandez-Moreira E. Merino M. Poza M. Mendez J. A. Soares N. C. (2011). Horizontal transfer of the OXA-24 carbapenemase gene via outer membrane vesicles: a new mechanism of dissemination of carbapenem resistance genes in *Acinetobacter baumannii*. *Antimicrob. Agents Chemother.* 55 3084–3090. 10.1128/AAC.00929-910 21518847 PMC3122458

[B85] Sabir S. Subramoni S. Das T. Black D. S. Rice S. A. Kumar N. (2020). Design, synthesis and biological evaluation of novel anthraniloyl-AMP mimics as PQS biosynthesis inhibitors against *Pseudomonas aeruginosa*. *Molecules* 25:3103. 10.3390/molecules25133103 32646050 PMC7412332

[B86] Saising J. Nguyen M. T. Hartner T. Ebner P. Bhuyan A. A. Berscheid A. (2018). Rhodomyrtone (Rom) is a membrane-active compound. *BBA-Biomembranes* 1860 1114–1124. 10.1016/j.bbamem.2018.01.011 29317198

[B87] Schaar V. Nordstrom T. Morgelin M. Riesbeck K. (2011). Moraxella catarrhalis outer membrane vesicles carry beta-lactamase and promote survival of Streptococcus pneumoniae and *Haemophilus influenzae* by inactivating amoxicillin. *Antimicrob. Agents Chemother.* 55 3845–3853. 10.1128/AAC.01772-10 21576428 PMC3147650

[B88] Seike S. Kobayashi H. Ueda M. Takahashi E. Okamoto K. Yamanaka H. (2020). Outer membrane vesicles released from aeromonas strains are involved in the biofilm formation. *Front. Microbiol.* 11:613650. 10.3389/fmicb.2020.613650 33488556 PMC7817658

[B89] Sharma A. K. Dhasmana N. Dubey N. Kumar N. Gangwal A. Gupta M. (2016). Bacterial virulence factors: secreted for survival. *Indian. J. Microbiol.* 57 1–10. 10.1007/s12088-016-0625-621 28148975 PMC5243249

[B90] Singh P. P. LeMaire C. Tan J. C. Zeng E. Schorey J. S. (2011). Exosomes released from *M. tuberculosis* infected cells can suppress IFN-gamma mediated activation of naive macrophages. *PLoS One* 6:e18564. 10.1371/journal.pone.0018564 21533172 PMC3077381

[B91] Sinha R. Howlader D. R. Ta A. Mitra S. Das S. Koley H. (2017). Retinoic acid pre-treatment down regulates V. cholerae outer membrane vesicles induced acute inflammation and enhances mucosal immunity. *Vaccine* 35 3534–3547. 10.1016/j.vaccine.2017.05.036 28545924

[B92] Su X. Wang H. Zhao J. Pan H. Mao L. (2011). Beneficial effects of ethyl pyruvate through inhibiting high-mobility group box 1 expression and TLR4/NF-kappaB pathway after traumatic brain injury in the rat. *Mediators Inflamm.* 2011:807142. 10.1155/2011/807142 21772666 PMC3136093

[B93] Tashiro Y. Toyofuku M. Nakajima-Kambe T. Uchiyama H. Nomura N. (2010). Bicyclic compounds repress membrane vesicle production and Pseudomonas quinolone signal synthesis in *Pseudomonas aeruginosa*. *Fems. Microbiol. Lett.* 304 123–130. 10.1111/j.1574-6968.2010.01897.x 20146747

[B94] Unal C. M. Schaar V. Riesbeck K. (2011). Bacterial outer membrane vesicles in disease and preventive medicine. *Semin. Immunopathol.* 33 395–408. 10.1007/s00281-010-0231-y 21153593

[B95] Vidakovics M. L. Jendholm J. Morgelin M. Mansson A. Larsson C. Cardell L. O. (2010). B cell activation by outer membrane vesicles–a novel virulence mechanism. *PLoS Pathog.* 6:e1000724. 10.1371/journal.ppat.1000724 20090836 PMC2799554

[B96] Volgers C. Benedikter B. J. Grauls G. E. Hellebrand P. H. M. Savelkoul P. H. M. Stassen F. R. M. (2017a). Effects of N-acetyl-L-cysteine on the membrane vesicle release and growth of respiratory pathogens. *Fems. Microbiol. Lett.* 364 10.1093/femsle/fnx087 28444395

[B97] Volgers C. Grauls G. E. Hellebrand P. H. M. Savelkoul P. H. M. Stassen F. R. M. (2017b). Budesonide, fluticasone propionate, and azithromycin do not modulate the membrane vesicle release by THP-1 macrophages and respiratory pathogens during macrophage infection. *Inflammopharmacology* 25 643–651. 10.1007/s10787-017-0359-357 28528362 PMC5671549

[B98] Wang Y. Hoffmann J. P. Chou C.-W. Höner Zu Bentrup K. Fuselier J. A. Bitoun J. P. (2020). Burkholderia thailandensis outer membrane vesicles exert antimicrobial activity against drug-resistant and competitor microbial species. *J. Microbiol.* 58 550–562. 10.1007/s12275-020-0028-21 32281050

[B99] Winter J. Letley D. Rhead J. Atherton J. Robinson K. (2014). *Helicobacter pylori* membrane vesicles stimulate innate pro- and anti-inflammatory responses and induce apoptosis in Jurkat T cells. *Infect. Immun.* 82 1372–1381. 24421041 10.1128/IAI.01443-13 PMC3993389

[B100] Yang J. Hwang I. Lee E. Shin S. J. Lee E.-J. Rhee J. H. (2020). Bacterial outer membrane vesicle-mediated cytosolic delivery of flagellin triggers host NLRC4 canonical inflammasome signaling. *Front. Immunol.* 11:581165. 10.3389/fimmu.2020.581165 33312172 PMC7708323

[B101] Yin B. Gu J.-D. Wan N. (2005). Degradation of indole by enrichment culture and *Pseudomonas aeruginosa* Gs isolated from mangrove sediment. *Int. Biodeter. Biodegr.* 56 243–248. 10.1016/j.ibiod.2005.10.001

[B102] Yu Y. J. Wang X. H. Fan G. C. (2018). Versatile effects of bacterium-released membrane vesicles on mammalian cells and infectious/inflammatory diseases. *Acta. Pharmacol. Sin.* 39 514–533. 10.1038/aps.2017.82 28858295 PMC5888691

[B103] Zhang W. Jiang X. Bao J. Wang Y. Liu H. Tang L. (2018). Exosomes in pathogen infections: a bridge to deliver molecules and link functions. *Front. Immunol.* 9:90. 10.3389/fimmu.2018.00090. 29483904 PMC5816030

[B104] Zhao T. Liu Y. (2010). N-acetylcysteine inhibit biofilms produced by *Pseudomonas aeruginosa*. *BMC Microbiol.* 10:140. 10.1186/1471-2180-10-140 20462423 PMC2882372

[B105] Zingl F. G. Kohl P. Cakar F. Leitner D. R. Mitterer F. Bonnington K. E. (2020). Outer membrane vesiculation facilitates surface exchange and in vivo adaptation of *Vibrio cholerae*. *Cell Host Microbe* 27 225–237.e8. 10.1016/j.chom.2019.12.002 31901519 PMC7155939

